# Hip pain and its correlation with cam morphology in young skiers—a minimum of 5 years follow-up

**DOI:** 10.1186/s13018-020-01952-8

**Published:** 2020-09-29

**Authors:** Josefin Abrahamson, Pall Jónasson, Mikael Sansone, Anna Swärd Aminoff, Carl Todd, Jón Karlsson, Adad Baranto

**Affiliations:** 1 Department of Orthopaedics, Institue of Clinical Sciences at Sahlgrenska Academy, University of Gothenburg and Sahlgrenska University Hospital, Gothenburg, Sweden; 2 Orthopaedic Research Unit, Sahlgrenska University Hospital, R-house, Level 7, 431 80 Mölndal, Sweden

**Keywords:** Cam morphology, Hip pain, Sports medicine, Follow-up study

## Abstract

**Background:**

There is conflicting evidence regarding the association between cam morphological changes and hip pain, and it remains unclear who with cam morphology will develop hip pain and who will not. This study aimed to investigate the correlation between cam morphology, hip pain, and activity level at a 5-year follow-up in young Alpine and Mogul skiers.

**Method:**

All students (*n* = 76) at Åre Ski National Sports High School were invited and accepted participation in this prospective study at baseline. Magnetic resonance imaging (MRI) of both hips was conducted to evaluate the presence of cam morphology (α-angle ≥ 55°) and its size alongside the reporting of hip pain, type, and frequency of training by the *Back and hip questionnaire*, at baseline. After 5 years, the skiers were invited to complete a shortened version of the same questionnaire.

**Results:**

A total of 60 skiers (80%) completed the follow-up questionnaire, of which 53 had concomitant MRI data. Cam morphology was present in 25 skiers (47.2%, 39 hips). Hip pain at baseline and at follow-up was reported in 17 (28.3%) and 22 (36.7%) skiers, respectively. No correlations were found between the activity level, the frequency, and the size of cam morphology and hip pain, except for the right hip α-angle at 1 o’clock and hip pain in skiers with cam morphology at baseline (*r*_*s*_ = 0.49; *P =* 0.03) and at follow-up (*r*_*s*_ = 0.47; *P =* 0.04). A total of 73.3% skiers had retired, of which 48% reported this was due to injuries.

**Conclusion:**

Hip pain was not shown to be correlated, or had a low correlation, with activity level and the presence and size of cam morphology in young skiers on a 5-year follow-up. Based on these results, cam morphology or activity level did not affect hip pain to develop during 5 years of follow-up in young skiers. Furthermore, this study highlights that almost 75% of young elite skiers had retired from their elite career with almost 50% reporting that this was due to injuries sustained from skiing.

## Introduction

Pain and discomfort from the hip and groin region are common in many athletes, with reported incidence varying depending upon the type of sport and level of activity [1–3]. Femoroacetabular impingement syndrome (FAIS) is a known cause of hip/groin pain in athletes [4, 5]. FAIS consists of a triad of hip pain, clinical signs (e.g. reduced hip range of motion (ROM)) and radiological findings [6]. The radiological findings of FAIS are present as an abnormal osseous deformity either at the femoral head-neck junction (cam morphology) and/or the acetabulum (pincer morphology). These deformities can generate a premature pathological abutment of the hip joint (particularly during hip flexion and/or internal rotation) and can potentially lead to labral and cartilage injuries [7–10]. Furthermore, there is strong evidence that cam morphology is a risk factor for osteoarthritis (OA) in the hip joint [11–13].

Athletes have a higher prevalence of cam morphology compared with general populations (48–79% vs. 5–55%) [14]. The prevalence of cam morphology in Alpine and Mogul skiers has been reported to be between 42 and 49% [15, 16]. Emerging evidence suggests a growing trend from high, repeated axial loading of the hip joints during skeletal maturation may be a risk factor for the development of cam morphology [17–20].

However, the association between cam morphology and hip pain has been questioned with conflicting levels of evidence being reported. Some studies have shown that a higher α-angle in individuals may be associated with hip pain and symptoms [5, 21, 22]. Furthermore, Khanna et al. reported a relative risk of 4.5 (95% CI, 2.3–9.1) to develop hip pain in the presence of cam morphology (α-angle ≥ 60°) in the general population [23]. In contrast, Mosler et al., van Klij et al., or Gosvig et al. found no conclusive association between cam morphology and hip pain or injury [1, 24, 25]. Additionally, the prevalence of cam morphology has been reported rather high (37%) in asymptomatic populations [26]. In patients with FAIS, arthroscopy is a common treatment with good postoperative outcomes [27]. However, it is still unclear who will develop hip pain, and if there are any potential predictors or timelines associated with this. Therefore, improving further knowledge around this issue will ultimately lead to the development of good prevention strategies in this area.

The aim of this study was to investigate the correlation between the presence and the size of cam morphology, hip pain, and activity level at a minimum of 5 years follow-up, in a cohort of young elite skiers.

## Methods

### Study population

**Fig. 1:**
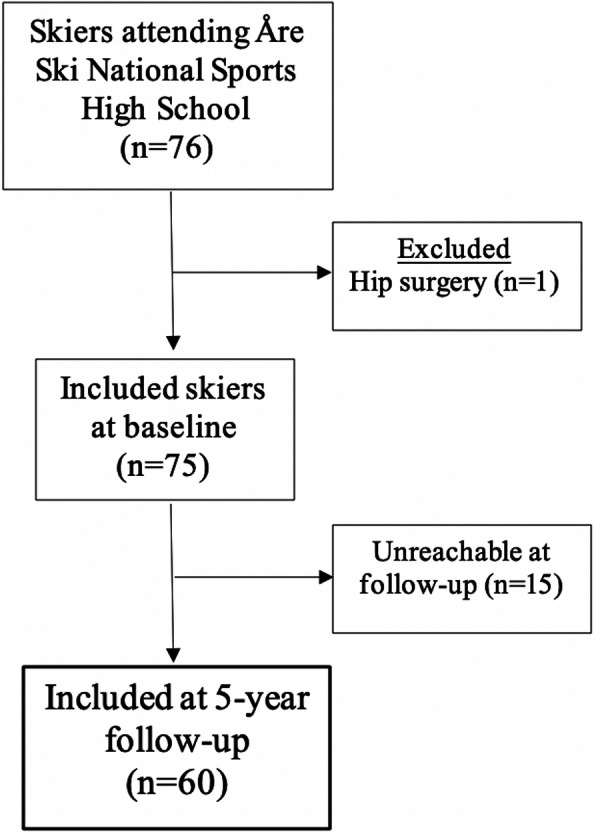
Flow chart of participants

All students (*n* = 76) at Åre Ski National Sports High school, grades 1–4 (16–18 years of age), were at baseline 2014 invited to participate in this prospective study. The exclusion criteria were related to any previously diagnosed injury or surgery to the hip and/or spine, or pregnancy. One subject had surgery for FAIS and was therefore excluded. Informed written consent was given by all individuals and by one parent, as the skier was younger than 18 years. In 2020, all subjects who participated at baseline were re-contacted by telephone and invited to participate in the 5-year follow-up. Fifteen (20%) were unreachable, while 60 skiers (80%) accepted to participate (Fig. 1).

### MRI examination

The participants underwent an MRI examination on both hips at baseline 2014. The MRI radial imaging was made with a GE Optima 450 Wide 1.5 T and a coil surface HD 8ch cardiac array was used. All MRI investigations were performed at the Radiological Department at Östersund Hospital, Östersund, Sweden.

#### The cam morphology

To evaluate the presence of cam morphology, the α-angle was measured as described by Nötzli et al. [28]. The α-angle measurement was taken from the MRI images and repeated in seven clockwise positions from the posterior (9 o’clock) to the anterior (3 o’clock) part of the femoral head-neck junction, according to Siebenrock et al. [29]. A cam morphology has previously been defined by an α-angle cutoff value of 55–60° [16–18]. In the present study, an α-angle of ≥ 55° at any of the seven positions was considered as the presence of cam morphology, and the size of cam morphology was defined with the size of the α-angle. As cam morphology have been reported to develop at the time of physeal closure [27, 29], whether the femoral growth plate was open or closed was examined as described by Siebenrock et al. [29]. Inter-observer test for the α-angle in this study cohort has previously been reported to be good with an ICC level of agreement of 0.75 [30].

### Self-reported hip pain

At baseline, all subjects were required to complete the *Back and hip questionnaire*, which has been used in previous studies [31–33]. The questionnaire assesses many hip and back pain parameters and specifically relates to the nature, location, onset, duration, and severity of pain, and includes the context of present and previous factors pertaining to daily living, work, training, and competition. This study focused upon the following questions: *Do you have, or have you had, hip pain*? (“yes, at present”; “previously not now”; “no never”); *Grade your hip pain* (“none,” “light,” “severe”); *Try to explain your hip pain* (“no pain, but stiffness”; “light aching” “severe aching/pain”: “sharp pain”); *How do you exercise/train at present?*; *How many days/week do you exercise?*; *Are you active in your main special sport?* (“yes, competing”; “yes, recreational/coaching”; “no”).

At the follow-up (between January and April 2020), the skiers completed a shortened version of the same questionnaire, verbally by phone. The shortened version included the same questions as abovementioned, and if the skiers answered with “yes, recreational/coaching” or “no” at the question *Are you active in your main special sport*?, an additional question regarding the reason why they were not still competing was asked.

Ethical approval was obtained by the Regional Ethical Review Board in Gothenburg at the Sahlgrenska Academy, Gothenburg University, Gothenburg, Sweden (ID number: 692-13).

### Statistical analysis

Descriptive statistics were described with mean and standard deviation for continuous variables and frequencies and percentage (%) for qualitative variables. Chi^2^ test or Fischer’s exact test was used for comparisons between categorical variables and independent *t* test for comparisons of continuous variables between the groups. To investigate the correlation between hip pain and the presence and size of cam morphology, Spearman’s rank-order correlation test (*r*_*s*_) was used. Values between 0.9 and 1.0 indicate very high correlation, 0.7 and 0.9 high correlation, 0.5 and 0.7 moderate correlation, 0.3 and 0.5 low correlation, and < 0.3 negligible correlation [34]. The paired *t* test or McNemar test was used to compare data between baseline and the follow-up. The Shapiro-Wilk test was used to test the normal distribution of data. The data were analyzed using IBM SPSS Statistics for Mac, version 26.0 (Armonk, NY: IBM Corp). To investigate the development of novel hip pain from baseline to the follow-up, a subgroup analysis was performed including skiers who were asymptomatic (i.e. answered “no never”) at baseline. All tests were two-sided with a level of *P* < 0.05 considered as significant.

## Results

A total of 60 skiers (mean age 23.6 years, 50% females) completed the follow-up questionnaire (answer rate 80%) at mean 5.7 (SD 0.5) years after baseline examinations. No differences were found between responders and those that had been lost to follow-up with regard to baseline age, gender, BMI, sport discipline (Alpine or Mogul), and the presence of cam morphology or hip pain. Table 1 shows the characteristics of the included skiers. One hip had poor imaging quality making it impossible for interpretation, and seven skiers failed to attend at the allocated time for the MRI examination, leaving 105 hips in 53 skiers with completed MRI examinations. All hips were shown with closed femoral growth plates and were considered to be skeletally mature. Cam morphology was shown to be present in 39 hips in 25 skiers (47.2%). In total, 17 skiers (28.3%) reported hip pain at present or past in either hip at baseline while 22 skiers (36.7%) reported the same at the follow-up, with no significant difference from baseline to the follow-up. Fourteen of the 22 skiers with hip pain reported stiffness as an explanation for their pain, two had undergone surgery for FAIS, one had known cam morphology, one snapping hip syndrome, and the remainder could offer no explanation for their hip pain (*n* = 4).

**Table 1:** Characteristics of included subjects at 5-year follow-up

No. of skiers, *n*	60
Age at follow-up, years^1^	23.6 (1.1)
Female gender	30 (50.0)
Ski-type at baseline	
Alpine	45 (75.0)
Mogul	15 (25.0)
Cam morphology (α-angle ≥ 55°)	
Either hip	25 (47.2)
Left hip only	19 (35.8)
Right hip only	20 (38.5)
Bilateral	14 (26.9)
Active at elite competitive ski level	
Yes	16 (26.7)
No	44 (73.3)
Hip pain at present or past	
Baseline	17 (28.3)
Follow-up	22 (36.7)

Values in number (%) unless specified

^1^Values in mean (SD)

Table 2 shows the comparison between those who were still active with elite competitive skiing (26.7%) at the time of follow-up and those who had finished their elite skiing career (73.3%), as well as reasons and the timescale for finishing their competitive careers. Almost every second skier (48%) reported an injury being the reason for quitting elite skiing, of which two (4.5%) underwent FAIS surgery. Training days per week at the follow-up did differ between the groups of still elite active and retired skiers (mean 5.3 vs. 3.8 days [*P* < 0.001]). No other differences or correlations were found between the activity level and hip pain (*r*_*s*_ ranged from − 0.05 to − 0.12) or cam morphology (*r*_*s*_ ranged from − 0.05 to − 0.11) at either baseline or the follow-up.

**Table 2:** Characteristics and comparisons of skiers still active and skiers retired at follow-up

	Active at elite competitive ski level		*P* value^1^
	Yes (*n* = 16)	No (*n* = 44)	
Age, years	23.6 (1.2)	23.6 (1.1)	n.s^2^
Female gender, *n* (%)	6 (38)	24 (55)	n.s
Cam morphology, *n* (%) hips	13 (43)	26 (35)	n.s
No cam morphology, *n* (%) hips	17 (57)	49 (65)	
Hip pain, *n*			
Baseline (yes/no)	5/11	12/32	n.s
Follow-up (yes/no)	8/8	14/30	n.s
Training days per week			
Baseline	5.9 (0.5)	5.8 (0.5)	n.s^2^
Follow-up	5.3 (1.0)	3.8 (1.6)^†^	< 0.001^2^
Years from graduation to finishing elite skiing		1.1 (1.4)	
Years from finishing elite skiing to follow-up		3.7 (1.5)	
Reason for quitting elite skiing, *n* (%)			
Hip injury		2 (4.5)	
Back injury		10 (22.7)	
Other injuries^a^		9 (20.5)	
Motivational		15 (34.1)	
Social		8 (18.2)	

Values in mean (SD) unless specified

^1^*χ*^2^ test

^2^Independent *t* test

^†^Significant difference between baseline and follow-up, paired *t* test [*P* < 0.001]

^a^Knee, 6; concussion, 3

### Hip pain vs. cam morphology

**Fig. 2:**
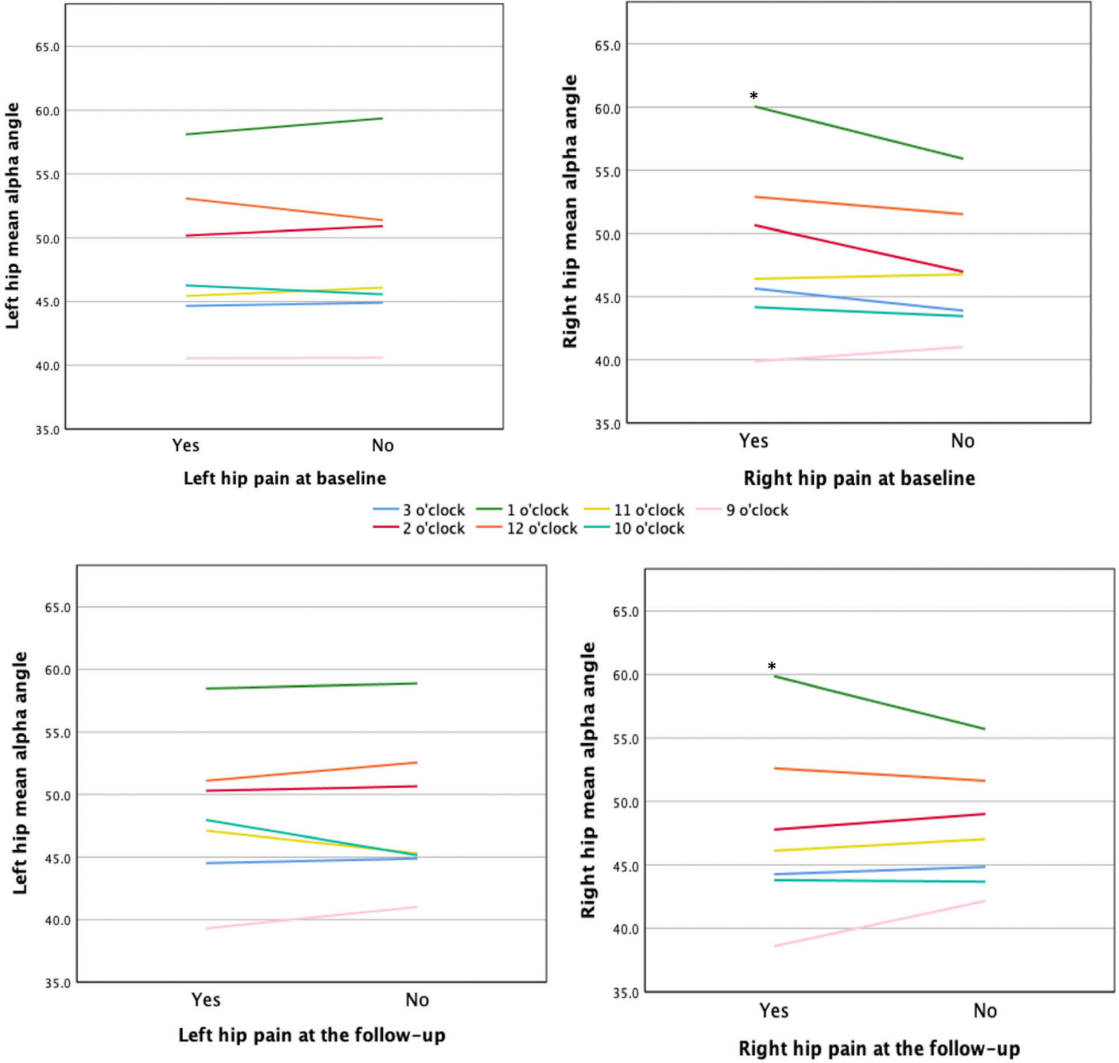
The mean α-angle in skiers with cam morphology, with and without hip pain. The mean α-angle in skiers with cam morphology (α-angle ≥ 55°) stratified by hip pain and no hip pain in the left hip at baseline (top left), right hip at baseline (top right), left hip at the 5-year follow-up (bottom left), and right hip at the 5-year follow-up (bottom right). *A significant correlation between hip pain and α-angle at *P* < 0.05, using Spearman’s rank-order correlation test

Reported hip pain did not change from baseline to follow-up in either those with cam morphology or those without. Fig. 2 shows the mean α-angle in skiers with cam morphology with and without hip pain at baseline and at follow-up, respectively. Significant correlations were found in skiers with cam morphology between the right hip α-angle measured at 1 o’clock and hip pain at baseline (*r*_*s*_ = 0.49; *P* = 0.03) and at follow-up (*r*_*s*_ = 0.47; *P* = 0.04). This was not seen in skiers without cam morphology. No other correlations were found between the presence or size of cam morphology, at any location, and hip pain at either baseline or follow-up.

#### Asymptomatic hips at baseline with MRI examination data

The subgroup analysis of those skiers with asymptomatic hips at baseline included 36 skiers and 71 hips. Eleven skiers reported hip pain at the follow-up, of which 8 had bilateral pain totaling 19 hips. Six of 19 painful hips had cam morphology (31.6%), whereas 16 of 52 asymptomatic hips (30.8%) had cam morphology. Neither the presence nor the size of cam morphology was shown to correlate with the development of hip pain at follow-up, except for the α-angle measured at 1 o’clock in the right hip of skiers that were shown to have cam morphology (*r*_*s*_ = 0.62; *P* = 0.03).

## Discussion

The main finding of this study has shown that self-reported hip pain was not significantly associated with the presence of cam morphology in young skiers with a minimum of 5 years follow-up. Although a significant correlation was shown for the right hip α-angle measured at 1 o’clock and hip pain in skiers with cam morphology, this should be viewed with caution as only a low correlation was shown to exist (*r*_*s*_ = 0.47 and 0.49). A secondary finding highlighted that nearly 75% (44 out of 60) had retired from skiing at an elite competitive level (at mean 1.09 years post-graduating the Ski National Sports High School) of which almost 50% reported were due to injuries.

The background to this study relates to the theory that FAIS may develop due to a combination of hip joint morphological changes, level of activity, and labral and/or cartilage damage alongside other yet unknown factors. The evidence appears conflicting regarding the relationship between cam morphology and hip pain. Similar to the results in the present study, van Klij et al. found inconclusive associations at a 5-year follow-up in young soccer players [24]. They found no associations between radiological based cam (α-angle ≥ 60), large cam (α-angle ≥ 78°), or visual cam (i.e. visually scored the shape of the femoral head-neck junction) and hip pain, while an association was found between visually scored large cam morphology and hip pain. Furthermore, a large cohort of 438 elite soccer players, investigated by Mosler et al., showed no correlations between hip injury/pain and cam morphology [1]. In contrast, Khanna et al. found in a prospective study a significantly larger α-angle in the hips that developed pain compared with pain-free hips, during 4 years of follow-up. Furthermore, they also reported a relative risk of 4.5 (95% CI, 2.3–9.1) for developing hip pain with an α-angle ≥ 60° [23]. Similar results were seen in the study by Larson et al. who studied 239 hips in 125 American football players. They found that pain was more commonly present in players with cam or mixed morphology, though only larger cam morphologies (increasing α-angle without specified cutoff value) were predictive for hip pain [5]. Furthermore, in a retrospective study by Guler et al., the α-angle was shown to be significantly higher in those with hip pain (55.9°) compared with those without (52.7°) [22]. An interpretation of this could mean that larger cam morphological changes might be painful earlier compared with smaller cam morphological changes that take longer to develop pain.

Although 47% of skiers were shown to have cam morphology in this study, the mean α-angle at each location (from 9 to 3 o’clock) was seen to be low (Fig. 2), and perhaps with a large mean α-angles, a greater correlation might have been found. In addition, this study included young skiers at baseline (mean age 17 years), resulting in a mean age of 24 years at the follow-up. It is possible that this may explain the low or absence of a correlation between hip pain and the presence and size of cam morphology in this study. Both Khanna et al. and Guler et al. included a general population with a mean age of 29.5 and 33.8 years, respectively [22, 23], and therefore had more years to develop hip pain. The duration of which cam morphology may have been present in this study might have been too short. In contrast, Larson et al. had a similar study population to the present study (young at the elite level) [5]. However, they used a retrospective study approach, evaluated hip morphology by plain radiographs, obtained pain from the school database, and did not exclude those with previous hip arthroscopy, which might explain the discrepancy.

The fact that almost 75% of the skiers were no longer at an elite level might also have affected the outcomes in this study. The majority of retired skiers reported skiing much less frequently with the additional total weekly training days being significantly less at the follow-up (3.8 vs. 5.8 days). This may suggest that the loading placed upon their hips, e.g. from less skiing, would have become reduced and therefore affecting the development of hip pain. In contrast, the presence of hip pain and cam morphology did not differ between those active elite skiers and those retired, while the number of training days per week did. This may suggest that the physical demands that skiing requires may not have a major role in hip pain, regardless of the presence of cam morphology. Furthermore, studies by both Guler et al. [22] and Khanna et al. [23] included cohorts from the general populations, and this may indicate that sporting participation may not be the only reason why an individual with cam morphology might develop hip pain. It still appears unclear why some athletes with cam morphology may function at the elite level for years, without secondary joint injuries, while others may not. The cam morphology is a known risk factor for the development of hip joint OA [11–13]. Therefore, understanding the development of hip pain related to cam morphology and preventing the possibility of hip OA through early identification is still of great interest.

Another important finding in the present study relates to the high percentage (almost 75%) of young skiers that had finished their elite careers due to injury with the mean 1.1 years from post-graduation. Comparing these results with those from other National Sports High Schools is difficult due to the lack of other similar studies. Previous studies have shown high injury incidence in athletes at National Sports High Schools, and 22–49% of these injuries being severe (i.e. time loss from training of 2 months or more) [3, 35]. Furthermore, a qualitative interview study by von Rosen et al. [36] showed that injured adolescent elite athletes did question the reasons to continue with elite sports and if elite sports were actually appropriate for them. In addition, a feeling of loneliness when all friends were training was highlighted, and it is possible that this might be more marked in National Sports High Schools where all students perform elite sports. An injury seems therefore not only to affect training and the ability to improve to the same degree as friends and competitors but may also affect a psychosocial state. The high proportion, in the present study, that reported finishing their elite careers before they had scarcely started might reflect this.

### Strengths and limitations

The strength of this study is the prospective study approach with a long follow-up time. The follow-up was made by phone, which has both its strengths and limitations. A strength is that incomplete or missing data/answers are minimized, while a possible answering impact from the caller might be seen as a limitation. To reduce the risk of misleading the responder or interpretation of answers, the person who made the follow-up phone call was blinded to baseline results and were strictly following the questionnaire. Fifteen skiers were lost to follow-up that may have affected the outcomes. However, baseline data of these and those included at the follow-up did not differ in any variable. The questionnaire used in this study has not been validated which is a limitation in itself. Furthermore, questions regarding past history do always include a risk of recall bias. The study group was set by the total number of students attending Åre Ski National High School at baseline and that was reachable at the follow-up. A larger sample size might have found larger differences between skiers with and without cam morphology. Other limitations may have included a lack of clinical examinations such as hip ROM, impingement signs, and MRI investigations to help identify other possible causes of hip pain (e.g., cartilage/labral/ligamentum teres damage). Perhaps, these additional investigations might have provided other reasons, such as microinstability of the hip, for the pain experienced by the skiers reporting pain in this study [37, 38]. However, the aim of this study was to investigate the correlation between hip pain and the presence and size of cam morphology based on the α-angle measurement, and as all skiers had closed femoral growth plates at baseline, a possible growth-related development of cam morphology may most likely not be present and affecting the outcome at the follow-up.

The clinical relevance of this study relates to the fact that almost three quarters of young elite skiers attending a National Sports High School had finished their elite career shortly after graduation (mean 1.1 years), with almost 50% reporting that this was due to injuries sustained from skiing.

## Conclusion

Activity level or the presence and size of cam morphology in young skiers does not correlate, or had a low correlation, with self-reported hip pain or that may have developed from baseline to 5 years of follow-up. Nearly 75% of the skiers had retired from their elite careers, with almost every second reporting this was due to different types of injuries.

## Data Availability

The datasets used and/or analyzed during this study are available from the corresponding author on reasonable request.

## Abbreviations

MRI: Magnetic resonance imaging
FAIS: Femoroacetabular impingement syndrome
ROM: Range of motion
OA: Osteoarthritis
